# Anderson–Fabry Disease: Red Flags for Early Diagnosis of Cardiac Involvement

**DOI:** 10.3390/diagnostics14020208

**Published:** 2024-01-18

**Authors:** Annamaria Iorio, Fabiana Lucà, Andrea Pozzi, Carmelo Massimiliano Rao, Cristina Chimenti, Stefania Angela Di Fusco, Roberta Rossini, Giorgio Caretta, Stefano Cornara, Simona Giubilato, Irene Di Matteo, Concetta Di Nora, Anna Pilleri, Sandro Gelsomino, Roberto Ceravolo, Carmine Riccio, Massimo Grimaldi, Furio Colivicchi, Fabrizio Oliva, Michele Massimo Gulizia, the Cardiac Rare Diseases Working Group Associazione Nazionale Medici Cardiologi Ospedalieri (ANMCO)

**Affiliations:** 1 Cardiology Department, Papa Giovanni XXIII Hospital, 24127 Bergamo, Italy; anita.iorio@hotmail.it; 2 Cardiology Department, Grande Ospedale Metropolitano, GOM, AO Bianchi Melacrino Morelli, 89129 Reggio Calabria, Italy; 3 Cardiology Department, Valduce Hospital, 23845 Como, Italy; 4 Department of Clinic, Internistic, Cardiovascular, Anesthesiologic and Geriatric Sciences, La Sapienza University of Rome, 00142 Rome, Italy; 5 Clinical and Rehabilitation Cardiology Department, San Filippo Neri Hospital, ASL Rome 1, 00135 Rome, Italy; 6 Cardiology Unit, Ospedale Santa Croce e Carle, 12100 Cuneo, Italy; 7 Levante Ligure Sant’Andrea Hospital, ASL 5 Liguria, 19121 La Spezia, Italy; 8 Arrhytmia Unit, Division of Cardiology, Ospedale San Paolo, Azienda Sanitaria Locale 2, 17100 Savona, Italy; 9 Cardiology Department, Cannizzaro Hospital, 95126 Catania, Italy; 10 Cardiology Unit, ASST Grande Ospedale Metropolitano Niguarda, 20162 Milano, Italy; 11 Department of Cardiothoracic Science, Azienda Sanitaria Universitaria Integrata di Udine, 33100 Udine, Italy; 12 Cardiology Brotzu Hospital, 09121 Cagliari, Italy; 13 Department of Cardiothoracic Surgery, Maastricht University, 6229 ER Maastricht, The Netherlands; sandro.gelsomino@maastrichtuniversity.nl; 14 Cardiology Unit, Giovanni Paolo II Hospital, 88046 Lamezia, Italy; 15 Cardiovascular Department, Sant’Anna e San Sebastiano Hospital, 81100 Caserta, Italy; 16 Cardiology Department, F. Miulli Hospital, Acquaviva delle Fonti, 70021 Bari, Italy; 17 Cardiology Department, Garibaldi Nesima Hospital, 95123 Catania, Italy

**Keywords:** Anderson–Fabry disease, cardiomyopathy, cardiac involvement, diagnostic red flags, left ventricular dysfunction

## Abstract

Anderson–Fabry disease (AFD) is a lysosome storage disorder resulting from an X-linked inheritance of a mutation in the galactosidase A (GLA) gene encoding for the enzyme alpha-galactosidase A (α-GAL A). This mutation results in a deficiency or absence of α-GAL A activity, with a progressive intracellular deposition of glycosphingolipids leading to organ dysfunction and failure. Cardiac damage starts early in life, often occurring sub-clinically before overt cardiac symptoms. Left ventricular hypertrophy represents a common cardiac manifestation, albeit conduction system impairment, arrhythmias, and valvular abnormalities may also characterize AFD. Even in consideration of pleiotropic manifestation, diagnosis is often challenging. Thus, knowledge of cardiac and extracardiac diagnostic “red flags” is needed to guide a timely diagnosis. Indeed, considering its systemic involvement, a multidisciplinary approach may be helpful in discerning AFD-related cardiac disease. Beyond clinical pearls, a practical approach to assist clinicians in diagnosing AFD includes optimal management of biochemical tests, genetic tests, and cardiac biopsy. We extensively reviewed the current literature on AFD cardiomyopathy, focusing on cardiac “red flags” that may represent key diagnostic tools to establish a timely diagnosis. Furthermore, clinical findings to identify patients at higher risk of sudden death are also highlighted.

## 1. Introduction

Anderson–Fabry disease (AFD), also known as Fabry disease, is an X-linked lysosomal storage disease characterized by the progressive and systemic accumulation of globotriaosylceramide (Gb3) in lysosomes, which may damage multiple organs [1].

Cardiac involvement frequently occurs in AFD patients, manifesting as left ventricular hypertrophy (LVH), conduction system impairment, and valvular abnormalities [2]. The severity of cardiac features has been related to adverse prognosis and sudden cardiac death (SCD) [2,3].

Notably, AFD-associated cardiomyopathy can be potentially reversible or stabilized after a specific treatment [4,5]. Thus, a timely diagnosis becomes crucial in improving the likelihood of a successful therapeutic strategy [6]. In this context, recognizing “red flags” can lead to the prompt detection of subclinical disease [1,7]. Furthermore, accurate and early identification of high-risk SCD patients is crucial in providing appropriate management [8].

However, due to the heterogeneous clinical presentations and multiple concealed clinical features, the diagnosis is challenging, and the underlying cardiac pathophysiology is incompletely understood.

The aim of this review is to discuss a reasoned diagnostic finalized for the early recognition and differential diagnosis of cardiac involvement in AFD.

## 2. General Features and Clinical Presentation of AFD

An AFD prevalence of 1/40,000–1/117,000 has been reported [2,9], although it appears to be underestimated [10,11,12,13]. Furthermore, a ten-year screening on 2034 probands with clinically suspected AFD significantly improved the rate of confirmed diagnosis [14], detecting 1.8% of GLA mutations [14].

The spectrum of disease severity is linked to the activity level of α-Gal A, which can range from deficiency to complete absence. A severe reduction in α-Gal A activity (<1% of mean normal) is associated with the classic form of AFD in hemizygous males, which is characterized by early clinical presentation, multiorgan involvement, more severe clinical manifestations, and adverse prognosis [6,15,16,17]. Males with a higher level of α-Gal A activity exhibit late-onset AFD with less severe disease expression than in the classic form [15,18]. The cardiac variant has also been identified within the non-classic forms, in which cardiac manifestation may be the exclusive or predominant disease expression. In these patients, differential diagnosis from other causes of LVH, such as cardiac amyloidosis (CA), hypertrophic cardiomyopathy (HCM), or hypertensive heart disease (HHD) is more challenging [19,20].

Since AFD follows an X-linked pattern of inheritance, affected females present with a slightly reduced to a near normal level of α-Gal A activity with the result that disease manifestation and prognosis are less severe than in their male counterparts. However, severe disease may also occur in females, who resemble the classic male phenotype of AFD patients [21]. Indeed, X-chromosome random inactivation (lyonization) leads to some cells expressing the normal allele and others with a mutated allele [21]. This leads to pleiotropic manifestations ranging from mild to severe disease expression.

Beyond the inheritance pattern and α-Gal A mutation, other factors such as genetic modifiers, environmental factors, and epigenetics may impact disease spectrum manifestation. These factors also contribute to inter and intra-familial variation [6].

**Figure 1:**
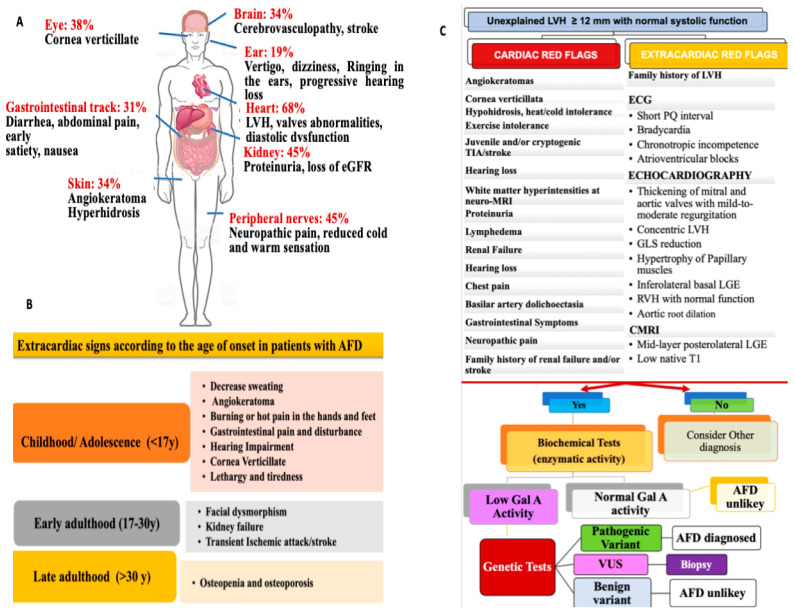
(**A**–**C**) Cardiac and extracardiac red flags: a diagnostic flowchart for making an AFD diagnosis. Abbreviation: LVH: left ventricular hypertrophy; GAL A: galactosidase A; AFD: Anderson–Fabry Disease; GLS: global longitudinal strain; RVH: right ventricular hypertrophy; LGE: late gadolinium enhancement; CMRI: cardiac magnetic resonance imaging; VUS: variant of unknown significance. (**A**) Potential systemic involvement, including neurologic signs and the gastrointestinal system, kidneys, and eyes. (**B**) Extracardiac signs according to the age of onset in patients with AFD. It is important to note that disease expression varies across different ages. Indeed, clinical symptoms can be divided into three consecutive age periods. Child and adolescent AFD patients present neuropathic involvement, acroparesthesia, and dyshidrosis symptoms. Gastrointestinal involvement with abdominal pain, diarrhea, nausea, and vomiting may also be associated with neurologic disturbance. Common signs are also represented by angiokeratomas and ophthalmological abnormalities. With few exceptions, these manifestations are more commonly described in both genders. Cerebrovascular disease, including stroke and transient ischemic attacks, may characterize the natural history of adult patients with AFD. Although the underlying mechanisms are not yet known, cardiogenic embolism, changes in the vessel walls, and abnormalities in coagulation pathway activation appear to be the leading causes of cerebrovascular complications. Renal disease and progressive kidney involvement represent significant causes of disease-related morbidity. Generally, renal failure affects adult patients with AFD, especially men who are in their 50s. Otherwise, affected male patients in the second or third decade of life may exhibit signs of early renal involvement, such as hyperfiltration, microalbuminuria, proteinuria, or isosthenuria (inability to concentrate urine). In female-affected patients, renal involvement is also clinically detectable, although it may be less severe than that observed in males. (**C**) The diagnostic work-up can be schematically divided into several steps. Biochemical tests are considered a first-line diagnostic tool in male patients. In contrast, a genetic test is required in females as a first-line step.

The heterogeneous clinical presentation of AFD often results in misdiagnosis. Extracardiac features should be considered for a timely AFD diagnosis [6,15] (Figure 1A). It is worth mentioning that disease expression varies across different ages (Figure 1B). The diagnostic workup of AFD should be based on a stepwise approach, including extracardiac and cardiac red flags, in order to recognize AFD as early as possible. (Figure 1C).

Indeed, differential diagnosis from other disorders manifesting with LVH is extremely relevant.

Specifically, recognizing red flags becomes crucial when systemic manifestations do not occur, considering the fact that all patterns of LVH may be present in AFD.

In this sense, standard ECG can be helpful in differentiating unexplained LVH forms. Particularly, the presence of LVH, with high QRS voltages and short PQ and PR intervals, should raise the suspicion of AFD. Moreover, this finding is unlikely to be present in other cardiomyopathies such as HCM (normal PQ-PR interval) or CA (low QRS complex) [22] (See Table 1).

Importantly, there is a certain degree of overlap between HCM and AFD, which may be considered a phenocopy of HCM. Therefore, some individuals with AFD are often misdiagnosed with HCM [22].

A prevalence of GLA gene mutations has been reported in approximately 1% of HCM patients, particularly in those with late-onset cardiac variants [23]. Similarly, among individuals undergoing surgical myectomy, genetic analysis revealed a GLA mutation in 1.3% of the patients. In light of these findings, a systematic screening for AFD in patients exhibiting the HCM phenotype should be performed. In cases of non-obstructive HCM, the LVH distribution can vary [24]. Significantly, some AFD patients with late-onset variants may have LVH that does not meet the 15 mm threshold for an HCM diagnosis. Indeed, the extent of LVH in HCM is generally more pronounced compared to AFD [24].

In HCM, late gadolinium enhancement (LGE) and impaired regional strain are typically observed in the most hypertrophic segments. In a study on 40 patients with LVH, including both AFD and HCM patients and matched for the degree of LVH and age, the FD group exhibited a lower left ventricular ejection fraction (LVEF), more reduced regional longitudinal strain (LS) in the inferolateral LV wall, and a more impaired right ventricular (RV) free wall LS [25]. Additionally, the pattern of hypertrophy was more frequently concentric in the FD group. Conversely, LVH is typically asymmetrical, affecting the septum in HCM [25].

Moreover, a higher LA enlargement degree and worse left atrial (LA) strain have been reported in HCM compared to AFD. Furthermore, a septal native T1 value < 1220 ms has been considered a helpful finding in differentiating FD from HCM with an accuracy of 95% [26].

Amyloidosis is a systemic disorder involving more than one organ, including the heart, kidneys, liver, and autonomic nervous system.

Immunoglobulin light chain (AL) amyloidosis and transthyretin (ATTR) amyloidosis have been recognized as the two predominant types of infiltrating amyloid. Hereditary transthyretin (TTR) amyloidosis is a result of a genetic mutation that predisposes individuals to the instability of the tetrameric structure of transthyretin [27]. In contrast, AL amyloidosis arises from the deposition of immunoglobulin light chains due to plasma-cell dyscrasia. An annual incidence of approximately 2500 to 5000 new cases has been estimated. AL amyloidosis is more frequent in men aged 65 years or older. Heart involvement is common, with >75% of subjects presenting cardiac symptoms. In CA, an accumulation of amyloid fibrils formed by misfolded proteins occurs in the interstitium of the heart. The heart conduction system is frequently affected due to progressive cardiac muscle dysfunction, which may lead to heart failure (HF) and cardiac arrhythmias [27].

Specific diagnostic tools, including bone scintigraphy, light chain assays, and tissue biopsies, are essential in confirming CA diagnosis. In native CA, T1 values and ECV increases have been described, with a global subendocardial or transmural LGE pattern [27].

In the context of echocardiographic assessment of CA, concentric LVH parameters such as posterior wall thickness (PWTd), interventricular septum thickness (IVSd), and relative wall thickness (RWT), along with a decrease in LS are considered characteristics [27].

## 3. Cardiac Involvement

### 3.1. Pathophysiology

Myocardial accumulation of Gb3 is crucial in developing AFD cardiomyopathy, although the accumulation of GB3 alone does not explain all-spectrum cardiac manifestations. The initial deposition of Gb3 characterizes the early phase of the disease. This occurs especially in and around the atrioventricular (AV) node, leading to early conduction abnormalities in AFD. Similarly, the infiltrative process is considered the main underlying mechanism of abnormalities in conduction tissue, such as sinus node disease and AV block. Beyond Gb3 accumulation, other pathophysiological pathways of storage-triggered mechanisms might explain the whole spectrum of AFD cardiac disease and progression [2]. Cardiac magnetic resonance imaging (CMRI) studies and experimental evidence support the inflammatory pathway as a pivotal mechanism in AFD progression. Indeed, progressive accumulation of Gb3 triggers inflammatory pathways that, in turn, lead to extracellular matrix remodeling and the release of hypertrophy-inducing growth factors.

These mechanisms have been suggested to have a pivotal role in developing overt cardiac structural manifestations [18,28], including LVH and diastolic dysfunction [29,30]. Later, the progressive inflammation with the tumor-grown Factor-B-mediated extracellular matrix activation also correlates with myocardial fibrosis and remodeling in the advanced stage of cardiac involvement.

### 3.2. Disease Manifestations: Patient Symptoms

Surveys and dedicated AFD registries lead to a unique opportunity to address cardiac clinical presentation [16,31,32,33]. Generally, cardiac symptoms have been reported with a higher prevalence in males than in females, increasing exponentially with age and disease progression for both genders [32]. Although cardiomyopathy is commonly asymptomatic during the early stage of AFD [31,32,34], index presentation as cardiac symptoms arises in almost 10% of patients. Otherwise, more than 60% experience HF, arrhythmias, angina, and syncope during the natural course of the disease [18,31,32]. The Fabry Outcome Survey (FOS) [35] and other selected AFD registries [5,16,31,32,36] have contributed to addressing cardiac manifestation in the affected population. However, the symptomatic spectrum of cardiac disease was reported at baseline registry enrolment, thus limiting knowledge about cardiac symptoms at the time of AFD diagnosis. Dyspnea and HF appear to characterize 22% of the AFD population [32]. Dyspnea seems to characterize all stages of the disease. In the early phase, patients with diastolic dysfunction [18,37] may exhibit dyspnea as a clinical-onset symptom. In contrast, in advanced stages, dyspnea is mainly related to systo-diastolic dysfunction, left ventricular hypertrophy (LVH) and valvular regurgitation, and overt HF [18,38].

Palpitations emerge as a clinical manifestation mostly related to supraventricular arrhythmias, with a higher prevalence in females than in males (21 vs. 15%) [32]. However, palpitations may also be a clinical manifestation of ventricular arrhythmias (VA), representing the most common life-threatening condition in AFD patients. As such, once AFD is suspected or diagnosed, the occurrence of palpitation should guide proper management. Likewise, syncope may suggest arrhythmias or left ventricular outflow tract obstruction (LVOTO), requiring careful attention. Indeed, syncope has been reported as the first clinical presentation of AFD, with prevalence ranging from 2 to 5.6% [18,28,32], wherein atrioventricular (AV) block and bradycardia along with LVOT appear as the main leading causes.

Chest pain is a common symptom with a similar prevalence between males and females [32]. However, its pathogenesis is still unclear. Higher myocardial oxygen demand secondary to LVH has been suggested as the main trigger. Further, the narrowing of intramural arteries due to hyperplasia and hypertrophy of GB3-engulfed smooth muscle cells along with coronary atherosclerosis has also been described as a possible cause of angina [39,40,41].

#### 3.2.1. Electrophysiologic Abnormalities and Arrhythmias Burden

**Figure 2:**
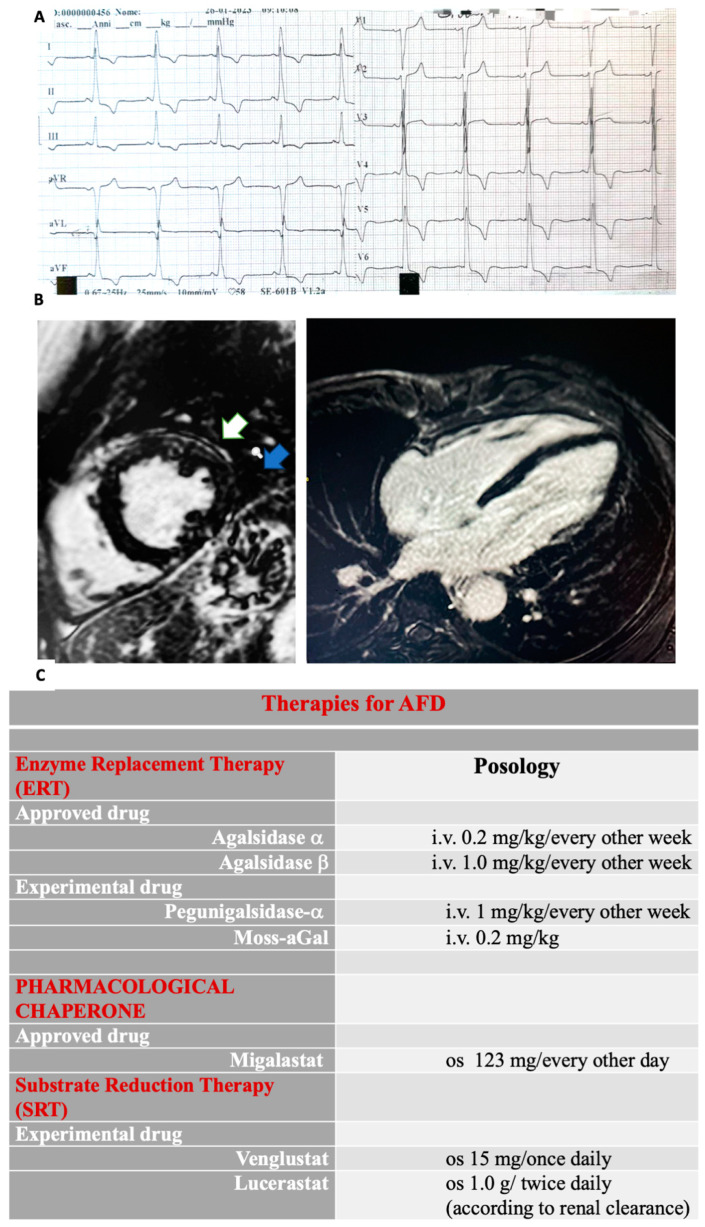
(**A**) Giant negative T-waves are shown in the ECG. (**B**) Cardiac magnetic resonance imaging (CMRI) short axis (**A**) and four-chamber (**B**) mid-basal inferolateral late gadolinium enhancement (LGE) in a patient with Anderson–Fabry disease. In the mid-antero-lateral wall, LGE is shown (white arrow). Intramyocardial LGE is shown in the mid-inferior-lateral wall (blue arrow). Recently, T1 mapping analysis has been proposed as a reliable tool for diagnosing AFD, demonstrating high sensitivity and specificity to discriminate this cardiomyopathy within a wide spectrum of conditions involving myocardial hypertrophy. (**C**) A timely multidisciplinary treatment consisting of both an FD-specific and a cardiovascular approach is crucial in order to avoid progression and irreversible systemic failure. ERTs and chaperone migalastat represent the approved disease-specific pharmacological strategies. Two different ERTs are used to treat AFD: algasidase alpha and algasidase beta. They both contain recombinant human a-Gal A and exhibit identical biochemical profiles. Nevertheless, different dosage regimens are used. ERTs have been shown to improve symptoms and reduce CV events and disease progression, particularly in both classic and cardiac forms, while in late-onset and advanced cardiac AFD cases, their effectiveness is poor. Since ERTs consist of recombinant human protein (a-Gal A), the development of neutralizing antibodies directed against the enzyme has been reported, particularly in males with classic AFD. Remarkably, the presence of antibodies against the enzyme decreases therapy efficacy. Algasidase alpha and algasidase beta have been approved in Europe and Canada, while in the USA, only algasidase beta use is allowed. Different studies have shown similar efficacy with no differences in the clinical event rates, although patients treated with algasidase beta were more likely to have a higher reduction in left ventricular mass (LVH). Moreover, less development of antibodies has been associated with algasidase beta than with algasidase alpha. In addition, chaperone therapy has been introduced to treat lysosomal storage disease. A small-molecule chaperone interacts with a mutant enzyme favoring its correct conformation, stability, and functioning. Migalastat, an oral pharmacological chaperone, has been recently proposed as an alternative to intravenous ERT in AFD. Its pharmacological action consists of stabilizing specific mutant (amenable) forms of α-Gal A in order to facilitate normal lysosomal trafficking. Moreover, other new therapies include second-generation ERTs and substrate reduction therapies (SRT). Gene and mRNA therapies are currently developing. Pegunigalsidase-a is a novel pegylated form of a-Gal A. Characteristically, its circulatory half-life is long-lasting. Moreover, heart and kidney uptakes are higher compared to current ERTs. Moss-aGalactosidase A (moss-aGal) is a moss-derived variant of human α-galactosidase. SRTs are another object of studies (venglustat is currently in phase II, whereas lucerastat is in phase III of clinical trials).

The electrocardiogram (ECG) remains an essential tool in the diagnostic assessment of AFD [22,42]. Various conduction abnormalities may be useful to detect cardiac involvement early. Therefore, the identification of ECG changes is crucial (Figure 2A). The early conduction manifestations are widely related to the accumulation of Gb3 affecting the conduction system [2]. Indeed, deposition of Gb3 around the AV node has been suggested as the earliest mechanism leading to an anomalous PR interval [43,44,45]. Notably, a short PR interval without a delta wave should increase the suspicion of AFD [44], often occurring before overt LVH development [14,45]. In this regard, interesting data has emerged from a study analysing conduction abnormalities in the ECGs of patients with newly diagnosed AFD without LVH. When these patients were compared with healthy controls, the PR interval was shorter in patients with early-stage FD (i.e., without LVH) than in the control group [45]. Interestingly, shortening of the P-wave duration was the main contributor to the shortened PR interval. Furthermore, the shortened P-wave duration resulted in a higher value than the PR interval to predict a diagnosis of AFD.

With AFD progression, later abnormalities include PR interval prolongation, voltage signs of LVH, and repolarization abnormalities [46]. Progressive disease, sinus, and AV node disease necessitate close monitoring for bradyarrhythmias, and the implantation of a pacemaker is often seen as part of the natural history of AFD patients [46].

Although the high prevalence of symptom-related arrhythmias (i.e., palpitations and syncope) along with the high incidence of permanent pacemaker implantations and sudden death suggest that arrhythmias may influence the long-term outcome of AFD, the exact prevalence of arrhythmias remains difficult to address.

Within restrictive pathophysiologic patterns characterizing AFD cardiomyopathy and other multiple factors, atrial fibrillation (AF) appears to be the most frequent supraventricular arrhythmias [18,28] and may occur as the first clinical presentation, even in young patients with AFD [47]. However, AF appears to be more frequent in affected patients aged > 50 years, with an incidence of 3.9% and 13.3% for persistent and paroxysmal AF, respectively [48].

Moreover, ventricular arrhythmias (VA) represent the most life-threatening condition and may also be detected as the first manifestation of AFD. Otherwise, no sustained VA is seen in patients with more advanced disease [16,28], occurring commonly in males with advanced stages and a moderate–severe LVH [48]. Furthermore, inflammation and fibrosis have been thought to play an essential role in determining the VA burden of AFD patients, sustaining an arrhythmogenic re-entry mechanism related to the myocardial fibrosis [28].

#### 3.2.2. Echocardiographic Findings

Echocardiography is an effective noninvasive tool for assessing structural and functional cardiac involvement in AFD [15,49,50]. LVH occurs in more than 50% and 20% of males and females, representing a key feature in AFD. The concentric pattern is the most common structural abnormality, although eccentric, asymmetric, and distal distributions have also been described [33,47]. Although LVH manifestation is usually delayed in females, a similar incidence has been reported in both genders [51]. Otherwise, depth echocardiographic analysis with targeted investigation aimed at excluding other causes of LVH should also be performed. This is especially relevant in cardiac AFD forms since LVH may be the only or predominant finding. In this clinical subset, evaluating cardiac and extracardiac clinical clues is essential in raising the suspicion of AFD-related LVH [44].

AFD often mimics hypertrophic cardiomyopathy (HCM) without left ventricular outflow tract obstruction (LVOTO). However, LVOTO, along with papillary muscle hypertrophy, may occur [51,52]. Indeed, resting LVOTO is rare, but it may be provokable during exercise in about 50% of patients with LVH related to AFD [53].

Right ventricular (RV) hypertrophy may also develop in nearly 25% of patients, with similar prevalence for both genders [33]. Ongoing valvular abnormalities may occur in both right- and left-sided valves, although mitral or aortic involvement appears to be more relevant [54]. Specifically, mitral valve (MV) involvement has been most commonly described in young patients, whereas aortic valve involvement occurs at an advanced age. Papillary muscle thickening and asymmetrical septal hypertrophy may accentuate mitral dysfunction, whereas aortic root dilatation may contribute to aortic regurgitation due to reduced leaflet coaptation [55]. Although valvular disease is common in AFD, few patients develop severe regurgitation or stenosis requiring cardiac surgery [33]

Pulsed wave Doppler and tissue Doppler imaging (TDI) are useful to assess subclinical diastolic and systolic dysfunction, which may occur before the development of overt LVH [56]. Diastolic dysfunction is related to increased ventricular stiffness and impaired relaxation due to intracellular Gb3 deposition and myocardial fibrosis TDI. The longitudinal or circumferential strain rate may reveal subclinical cardiac involvement before the onset of LVH or systolic/diastolic impairment. However, TDI remains unspecific and poor in discriminating AFD from other cardiomyopathies. Conversely, the loss of the base-to-apex circumferential strain gradient seems to be the most characteristic echocardiographic pattern in AFD cardiomyopathy [57]. This parameter, in a cohort of 77 patients (*n* = 37 with AFD cardiomyopathy, of whom 57% had LVH; *n* = 27 with HCM; *n* = 19 healthy subjects), identified AFD cardiomyopathy irrespective of the presence of LVH [57]. Severe LV systolic dysfunction remains uncommon and usually develops in advanced stages [58]. Importantly, when LV systolic dysfunction occurs, AFD patients show a higher risk of HF-related mortality [59]. Moreover, LV systolic dysfunction, hypokinesis, and thinning of the base of the LV posterior wall appear to be echocardiographic findings that point out HF progression [60].

#### 3.2.3. Cardiac Magnetic Resonance Imaging Findings

CMRI is considered the gold standard for assessing LVH and myocardial fibrosis in AFD [61,62]. Remarkably, LGE in the subepicardial basal–mid-inferolateral wall is a hallmark of AFD cardiomyopathy. This is especially relevant in advanced-stage disease [62] (Figure 2B). T1 mapping is a well-established CMR technique used for assessing myocardial tissue characteristics and detecting myocardial edema, accumulation of intra-myocyte lipids, and expansion of extracellular volume, which may involve proteins or iron deposition [63,64].

It involves the measurement of the quantitative T1 signal originating from the myocardial tissue, which is then subjected to post-processing to generate a color-coded map representing the myocardium. Particularly, native T1 mapping evaluates the intrinsic myocardial longitudinal relaxation time without needing a contrast agent [63,64].

A low native T1 value is considered an indicator for identifying myocardial glycosphingolipid accumulation before the development of LVH, allowing a timely identification of cardiac impairment during a pre-hypertrophic phase [65]. Importantly, the reduction in T1 values during the pre-LVH stage has been correlated with a decrease in global longitudinal strain (GLS) [65].

Moreover, it has been recognized that, among patients with confirmed LVH, the utilization of T1 provides the distinction of AFD from other prevalent primitive and secondary forms of LVH [63,66] when applying a predetermined cut-off [67].

Therefore, it has been demonstrated that a decreased T1 value within the context of LVH has a remarkable sensitivity and specificity for the recognition of AFD, enabling the differentiation of this condition from other hypertrophic forms where T1 values remain within the normal or elevated range, including hypertrophic cardiomyopathy (HCM), AL amyloidosis, hypertensive heart disease, severe aortic stenosis [66].

Sado et al. [67] studied 227 subjects (44 AFD, 34 HCM, 21 severe AS, 20 cardiac amyloidosis, 41 adults with hypertension, and 67 healthy volunteers).

In comparison to the healthy volunteers, septal T1 values were lower in individuals with AFD and higher in those with other pathological conditions; among patients with LVH, T1 values provided complete differentiation between AFD and other diseases, with no overlapping values. In AFD patients, T1 values exhibited an inverse correlation with wall thickness and were abnormal in 40% of subjects without LVH. Importantly, AFD patients showed a pseudo normalization or elevation of T1 values in the LV inferolateral wall, which correlated with the presence or absence of LGE.

Pica et al., in a study on 63 AFD patients who underwent a comprehensive assessment, including CMR, ECG, and echocardiography, demonstrated that in the 40% of individuals with AFD but without LVH, lower native T1 values were associated with reduced global longitudinal (GL) strain values, evaluated with speckle tracking echocardiography (STE), and early diastolic function impairment, suggesting that a reduction in T1 values, occurring prior to the development of LVH, is linked to early diastolic and systolic abnormalities [67,68].

Moreover, because fibrosis is typically absent in the septum of the majority of AFD patients with LVH, in contrast to other forms of LVH, a T1 value reduction is not counteracted by any potential T1 prolongation, which could be caused by fibrosis [67]. Consequently, among patients with LVH who undergo CMRI, the identification of a reduced T1 value should lead to referring the individual for specialized testing to detect AFD [63,69].

However, it is worth noting that in the presence of normal native T1 values, AFD cannot be excluded due to the fact that, although rarely, individuals with mild LVH, particularly women or those in advanced phase, might have a pattern of pseudonormalization with apparently normal native T1 values [63,69] when both sphingolipid deposition and fibrosis coexist.

A study involving 44 AFD patients revealed a pattern of pseudonormalization or elevation of T1 values, specifically in the LV inferolateral wall, which were correlated with the presence of late LGE in the same area of the heart [67].

A study on 182 individuals with AFD (167 adults and 15 children) revealed that in children, T1 values consistently remained within the normal range, although they have been observed to decrease with age. In the overall cohort, the reduction in T1 values associated with aging was more significant and pronounced in males [70]. Notably, LVH, LGE, and ECG abnormalities were observed at earlier stages in males. Notably, LVH increased particularly in women; however, T1 values and LVH became less correlated, differently from men, who revealed a correlation between the increase in T1 values and the progression of LVH [70].

Therefore, T1 mapping analysis has been confirmed as a reliable diagnostic tool in AFD irrespective of sex, ventricular function, or hypertrophy morphology [67,71], further studies are needed to confirm its role in this cardiomyopathy.

## 4. Diagnostic Workup: The Roles of Genetic and Biochemical Testing, Biopsy, and Biomarkers in AFD

A definitive diagnosis should rely on genetic testing, enzyme activity, and tissue studies (whenever possible) showing Gb3 accumulation (Figure 1C). [1,14].

Specifically, biochemical measurements of α-Gal A activity in the blood and leukocytes occur through the detection of the plasma levels of the storage product GB3 and its degradation product (Lyso-GB3). For males with the classic form, in whom α-Gal A activity is severely reduced or absent, a biochemical test is often sufficient for diagnosis. In this clinical context, the assessment of α-Gal A enzyme activity should be performed as a first-line test. However, in rare cases, male patients might have residual α-Gal A activity, and this limits the diagnostic ability of the biochemical α-Gal A test. Genetic mutation research becomes necessary for these patients to diagnose [6]. Similarly, in females with AFD and normal or slightly deficient activity of α-Gal A, diagnosis requires a genetic test as the initial approach [72,73].

Otherwise, for all AFD patients, genetic testing increases the diagnostic utility of the biochemical test. Indeed, different Gal A mutations are associated with a different spectrum of α-Gal A activity and disease manifestations. Particularly, non-sense, consensus splice site, and frameshift mutations are often related to lower or no α-Gal A enzyme activity. They are usually associated with the classic form of AFD. In contrast, missense mutations and rare cryptic splicing mutations can be associated with residual α-Gal A enzyme activity characterizing the late-onset phenotypes [6,15,74].

When a challenging interpretation of GLA mutations or starting therapy is controversial, an endomyocardial biopsy (EMB) may be required [75,76], providing definitive evidence of AFD by showing fine-granulated vacuolization through Sudan-black staining, concentric lamellar bodies formed by Gb3, and typical lysosomal inclusions or “zebra” bodies via electron microscopy. The vacuolation and the presence of lamellar bodies, revealed by light and electron microscopy, respectively, have been considered histological characteristic findings [2,64].

The latest European Society of Cardiology (ESC) guidelines [69] have recommended the use of EMB, highlighting that it could also be performed in other involved organs such as skin and renal tissue. Importantly, it should be taken into account that administering some drugs might induce drug-induced phospholipidosis with an intracellular accumulation of phospholipids in different tissues mimicking zebra bodies [77,78]. Moreover, the role of a multidisciplinary approach and the importance of expert pathologists has been pointed out [69].

Over the last years, the assessment of the deacylated form of Gb3 has been suggested as a reliable biomarker for AFD [79,80]. Indeed, in a cohort of 2360 patients with clinical symptoms suggestive of AFD, high levels of lyso-Gb3 were associated with both the classic and the non-classic forms [79]. However, further studies are required to confirm the reliability of this marker.

Furthermore, persistently higher levels of high-sensitivity troponin T (hs-TNT) have been reported in more than 21% of AFD patients. Notably, a significant association has been documented between troponin concentrations and LGE in AFD [81].

Additionally, hs-TNT may be helpful for staging and monitoring AFD progression, playing an important role in the follow-up of individuals with AFD [81]

Moreover, elevated serum values of N-terminal pro-brain natriuretic peptide (NT-proBNP) have been observed in patients with cardiac abnormalities in AFD, correlating with symptom severity and echocardiographic indicators of a higher left ventricular (LV) filling pressure [82]. Notably, an increased concentration of NT-proBNP has also been reported in patients lacking echocardiographic evidence of LVH. This implies that measuring NT-proBNP could be helpful for an early disease’s detection, identifying individuals in subclinical stages [82].

## 5. Therapy

Disease-specific treatments for reducing CV events, such as enzyme replacement therapy (ERTs) [83,84] and the pharmacological chaperone migalastat [85,86] have been recently approved, while emerging molecules are developing. ERTs have dramatically improved the quality of life of AFD patients, reducing neuropathic pain, gastrointestinal symptoms, and CV events. ERTs prevent LVH development and favor LVH regression in the initial stage in patients with both classic and cardiac forms, exerting effects on cardiac structure, including a gradual reduction in the interventricular septum (IVS) thickening and a decrease in the left ventricle mass index (LVMi) [83,84,87,88]. Conversely, in the late-onset cardiac forms and in advanced cardiac cases, its efficacy is poor, without evidence of an effect on myocardial fibrosis and LVH [6,89,90,91,92].

Several determinants that may lead to a reduced ERT response have been reported [83,84].

Data from a cohort of 32 AFD patients over a three-year period demonstrated that, among patients who did not exhibit fibrosis, ERT with recombinant α-galactosidase led to a substantial decrease in LVM, an enhancement in myocardial function, and an increased exercise capacity. Conversely, patients with mild or severe fibrosis experienced only a modest reduction in LVH, with no discernible improvement in myocardial function or exercise capacity [93]. These results strongly suggest that initiating ERT prior to the development of myocardial fibrosis is the optimal approach for achieving sustained long-term improvements in myocardial structure and function, as well as exercise capacity [93]. Data from the Fabry Registry on 163 AFD males, 115 treated with agalsidase-β and 48 untreated, revealed that the administration of agalsidase-β for a duration of at least two years may lead to an improvement or stabilization of LVM in males with AFD. Indeed, untreated patients had a 3.4-fold higher risk of experiencing a more rapid increase in LV mass compared to those who were treated. Additionally, individuals aged 40 years or older were more likely to experience LVH progression compared to those under the age of 30 years [94].

A meta-analysis encompassing 15,305 participants showed that the use of agalsidase beta was linked to a notably reduced occurrence of renal, cardiovascular, and cerebrovascular events when compared to individuals not receiving ERT [95].

A significant reduction in LVMi after 12 months of treatment with agalsidase beta has been shown in a study involving nine AFD patients [88].

Accordingly, Weidemann et al. conducted a study on AFD in sixteen patients treated with agalsidase beta and followed up with for 12 months, demonstrating a reduction in LVH accompanied by an improvement in LV function [96].

Additionally, small-molecule chaperones’ use has been introduced for treating lysosomal storage disease [97]. Migalastat, an oral chaperone, favors enzymatic stabilization of the specific mutant α-Gal A variant (amenable forms) [85,86], seeming to reduce LVH, renal, and cardiovascular events [98] (Figure 2C). Particularly, an improvement in LVMi has been shown in the majority of patients treated with migalastat [98,99,100,101]. The randomized trials FAMOUS, FACETS, and ATTRACT demonstrated a significant reduction in LVMi after 24 and 30 months of therapy [98,99,102,103].

## 6. Future Directions

AFD is a progressive disease developing into end-stage organ disease and death.

A timely diagnosis is paramount to guide early therapy and the proper management of patients and family members. Due to pleiotropic manifestations, a systematic approach is needed to increase the success of diagnosis. For this goal, an accurate family history collection and extra-cardiac assessment may identify “clinical pearls” to raise suspicion of AFD. Likewise, a depth cardiac evaluation aimed at identifying hallmarks of heart disease related to AFD remains crucial in raising suspicion of this disease.

Particularly, since AFD usually manifests as LVH, a differential diagnosis from other cause of LVH become mandatory. When AFD is suspected, an appropriate initial approach to biochemical and genetic tests plays a crucial role in diagnosing. Accordingly, a different diagnostic impact of these tests should be taken into account within both genders. After diagnostic confirmation, cascade family genetic screening for X-linked inheritance is recommended. Furthermore, careful attention to the early identification of the clinical manifestation of disease in the family carrier should be performed. For all patients, prognostic risk stratification and accurate staging of cardiac involvement are highly advisable beyond diagnostic assessment to identify findings such as LVH, LV dysfunction, late gadolinium enhancement, and arrhythmia burden.

Cardiovascular diseases (CVD) have been reported as the leading cause of death in AFD, both in males and in females [16,18,28]. Specifically, data from the registry showed a 75% prevalence of cardiovascular death, whereas SCD accounted for around 60% of reported deaths [8].

Although well-known clinical features characterizing advanced cardiac involvement have been identified, no models for the risk prediction of SCD exist. Further data identifying risk factors for SCD and VA are limited [8]. Most of the current knowledge of risk stratification is derived from a systematic review of 13 studies, wherein male sex, age > 40 years, the presence of LVH, late gadolinium enhancement, and VA were associated with a higher risk of SCD [8]. Thus, clinicians might consider these results to identify those patients with a higher risk of life-threatening arrhythmias.

Although specific treatments have been associated with reductions in LV mass, HF, and VA and with an improvement in myocardial function [84,98,104], no data concerning the impact of medical treatment on SCD are available. Future studies addressing this topic should be encouraged.

## 7. Conclusions and Future Perspectives

AFD is X-linked lysosomal storage masqueraded by multiple cloaks. Timely diagnosis and early risk stratification remain essential to benefit from the current therapeutic approach and management. In this context, a complete collaboration of cardiologists and other specialists becomes essential to shed light on the future better management of this complex disease.

## Figures and Tables

**Table 1:** ECG/biochemical/clinical findings for differential diagnosis from other causes of LVH.

	AFD	CA	HCM
ECG	PR short Q (<40 ms) ↑ Sokolow–Lyon index > amyloidosis QTc < 440 ↑ Inferior ST-depression HCM	↓ QRS voltages (discrepancy between QRS voltages and LV mass) QRS voltage ≤ 0.5 mV (limb lead) QRS voltage ≤ 1 mV in precordial lead Sokolow–Lyon index < 1.5 mV Pseudo-infarct pattern (Q waves on two contiguous leads in the absence of CAD)	Pathological Q-waves Deep S-waves in V1–V3 High R-waves in V4–V6 with abnormal T-waves Giant symmetric negative T-waves (apical HCM). ST segment elevation in anterior leads (pseudo-STEMI pattern) Mild ST–T-wave modifications Diphasic T-waves Inverted T-wave in aVL in inferior and lateral leads Isolated inverted T-wave in aVL
Biochemical	NT-proBNP, BNP MRproANP, MMP2 MMP9 Galectin-1, Galectin-3	cTnTM cTnI NT-proBNP	BNP NT-proBNP ANP
Clinical	Family history of AFD or Cornea verticillata Anydrosis or hypohidrosis Personal or family history of renal failure Angiokeratome Personal or family history of acroparesthesias Personal or family history of heat or cold intolerance	Renal dysfunction proteinuria Carpal tunnel Orthostatic hypotension Peripheral neuropathy Gastrointestinal disorders Hypothyroidism Lumbar spinal stenosis Spontaneous biceps tendon rupture Ocular floaters Macroglossia and periorbital purpura (pathognomonic for AL amyloidosis)	Diastolic dysfunction Obstruction to LVOT Cardiac arrhythmias Chest pain Dyspnea Exercise intolerance Orthopnea Peripheral edema HFpEF Palpitations Presyncope Syncope

AFD: Anderson–Fabry disease; CA: cardiac amyloidosis; HCM: hypertrophic cardiomyopathy; NT-proBNP: N-terminal pro-B-type natriuretic peptide; BNP: B-type natriuretic peptide; MRproANP: mid-regional pro-atrial natriuretic peptide; MMP: matrix metalloproteinases; cTnT: cardiac troponin T; cTnI: cardiac troponin I; LVOT: left ventricular outflow tract obstruction.
